# Face Alignment in Thermal Infrared Images Using Cascaded Shape Regression

**DOI:** 10.3390/ijerph18041776

**Published:** 2021-02-12

**Authors:** Kent Nagumo, Tomohiro Kobayashi, Kosuke Oiwa, Akio Nozawa

**Affiliations:** Graduate School of Science and Engineering, Aoyama Gakuin University, Kanagawa 252-5258, Japan; nagumo@ieee.org (K.N.); kobachi369@gmail.com (T.K.); oiwa@ee.aoyama.ac.jp (K.O.)

**Keywords:** face alignment, thermal infrared image, facial thermal image, cascaded shape regression, real-time measurement, remote measurement

## Abstract

The evaluation of physiological and psychological states using thermal infrared images is based on the skin temperature of specific regions of interest, such as the nose, mouth, and cheeks. To extract the skin temperature of the region of interest, face alignment in thermal infrared images is necessary. To date, the Active Appearance Model (AAM) has been used for face alignment in thermal infrared images. However, computation using this method is costly, and it has a low real-time performance. Conversely, face alignment of visible images using Cascaded Shape Regression (CSR) has been reported to have high real-time performance. However, no studies have been reported on face alignment in thermal infrared images using CSR. Therefore, the objective of this study was to verify the speed and robustness of face alignment in thermal infrared images using CSR. The results suggest that face alignment using CSR is more robust and computationally faster than AAM.

## 1. Introduction

A method for remotely evaluating physiological and psychological states based on facial skin temperature measured by infrared thermography has attracted considerable interest. Biological information is used in various fields, such as medicine, welfare, and industry. In general, the measurement of biological signals often requires physical restraint, and the measurement itself may cause mental or physical stress to the subject [1]. Conversely, infrared thermography can conduct contactless, non-invasive skin temperature measurements with high sensitivity, accuracy, and reproducibility [2,3,4,5]. In a thermal environment that is windless and non-sweat inducing, the main cause of variation in skin temperature is skin blood flow [6]. Since the autonomic nervous system controls skin blood flow as a part of the circulatory system’s function to regulate body temperature, skin temperature has been used to assess the activity of the autonomic nervous system [7]. For this assessment, facial thermal infrared images are particularly suitable for application because the face is often exposed and unobstructed by clothing. Many previous studies have been conducted on the estimation of physiological and psychological states based on facial skin temperature distribution [8,9]. For example, studies have been conducted that estimate vital data such as respiratory rate [10] and heart rate [11], sleepiness [12,13,14], emotions [15,16,17], mental stress [18,19], and anomaly detection in facial skin temperature distribution [20]. These previous studies used the temperature distribution of the entire face or the temperature of specific Regions of Interest (ROI), such as the nose, mouth, and cheeks for evaluation. Therefore, it is important to automatically detect faces and facial landmarks from thermal infrared images with high accuracy and stability. In recent years, infrared thermography has lowered in price but has bettered in performance. The resolution of thermal infrared images has increased, and it is possible to measure multiple people with a single thermal infrared image. Therefore, in face detection and detection of facial landmarks in real environments, it is desirable to increase the speed to analyze multiple people at once.

The Active Appearance Model (AAM) [21] is one of the most popular methods for automatically detecting facial landmarks in thermal infrared images. AAM statistically models the changes in face shape and overall facial appearance and aligns the face shape with the model through nonlinear optimization. Kopaczka et al. conducted face alignment in thermal infrared images using AAM based on intensity, Histogram of Oriented Gradients (HOG), and Dense Scale Invariant Feature Transform (DSIFT) features [17,22]. However, in general, AAM is expensive because it solves an exact optimization problem. It also suffers from low robustness to poses, illumination, facial expression changes, and unknown subjects that are not included in the training set [23].

To solve these problems, Cascaded Shape Regression (CSR) has been proposed for face alignment in visible images [24,25,26,27]. In the CSR approach, facial landmark detection is estimated by regression, and the solution is updated multiple times by a multi-stage estimator to detect the facial landmarks. Face alignment using CSR is highly real-time. Ren et al. [26] reported face alignment at more than 3000 FPS speed. Hence, it is expected that CSR can be used for faster face alignment in thermal infrared images. However, no studies have been reported on face alignment in thermal infrared images using CSR. Therefore, the objective of this study was to verify the speed and robustness of face alignment in infrared images using CSR. First, a CSR model was created. Next, we trained and evaluated the CSR on the thermal infrared images acquired in our experiments. The results suggest that face alignment using CSR is more robust and computationally faster than AAMs proposed in the previous study, which is reported in this paper.

## 2. Cascaded Shape Regression

If $x_{i},y_{i}$ are the x,y coordinates of the *i*th facial landmark, then the face shape vector represented by the *M* facial landmarks is $S={\left[x_{1},y_{1},\cdots ,x_{M},y_{M}\right]}^{T}$. The cascaded shape regression model is a model with a multi-stage structure estimator with *T* number of stages, which predicts the face shape $S^{\left(t\right)}$ in a cascaded manner. Given the initial face shape $S_{0}$ and the input image I, the CSR model is updated by the estimator to find the shape difference fraction $\Delta S^{\left(t\right)}$ and update the solution. At stage *t*, $S^{\left(t\right)}$ and $\Delta S^{\left(t\right)}$ are regressed as follows:

(1) $$\begin{array}{c} S^{\left(t\right)}=S^{\left(t-1\right)}+\Delta S^{\left(t\right)}, \end{array}$$

(2) $$\begin{array}{c} \Delta S^{\left(t\right)}=r^{\left(t\right)}\left(I,S^{\left(t-1\right)}\right) \end{array}$$

where t∈1,⋯,T is the number of estimators corresponding to each stage of the CSR, and $r^{\left(t\right)}$ is the estimator. The loss function is represented as follows:

(3) $$\begin{array}{c} \underset{r^{\left(t\right)}}{arg min}\sum_{i=1}^{N}{\left|\hat{S_{i}}-\left(S_{i}^{\left(t-1\right)}+r^{\left(t\right)}\left(I_{i},S_{i}^{\left(t-1\right)}\right)\right)\right|}^{2} \end{array}$$

where ${\hat{S}}^{\left(t\right)}$ is the ground’s true face shape, and *N* is the number of images for training. In the CSR, training is performed in such a way that this loss function is minimized. In this study, we estimated facial landmarks using the ensemble of regression tree learning methods used by Vahid et al. [27]. Gradient boosting was used as the training estimator. At each split node of the regression tree, the intensity difference sentence feature of two pixels [25,28] is determined based on the threshold. To train each split node, 400 randomly sampled features were computed.

## 3. Experiments

### 3.1. Experimental Methods

**Figure 1:**
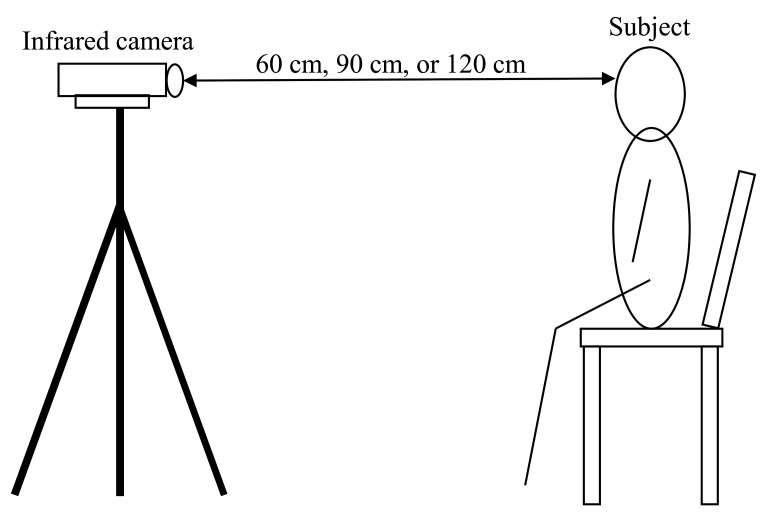
Experimental system. The distance between the subject and infrared camera was set at three different distances: 60 cm, 90 cm, or 120 cm.

**Figure 2:**
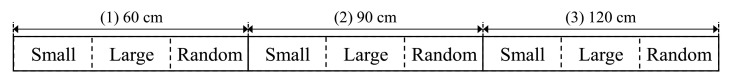
Experimental protocol.

**Figure 3:**
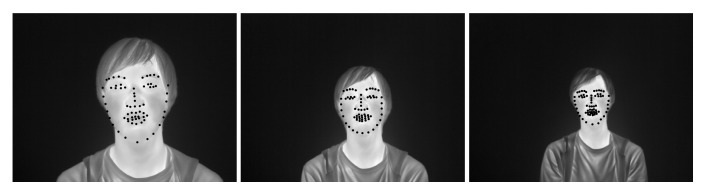
Examples of thermal infrared images acquired under three different distance conditions. From left to right: 60 cm, 90 cm, and 120 cm.

**Figure 4:**
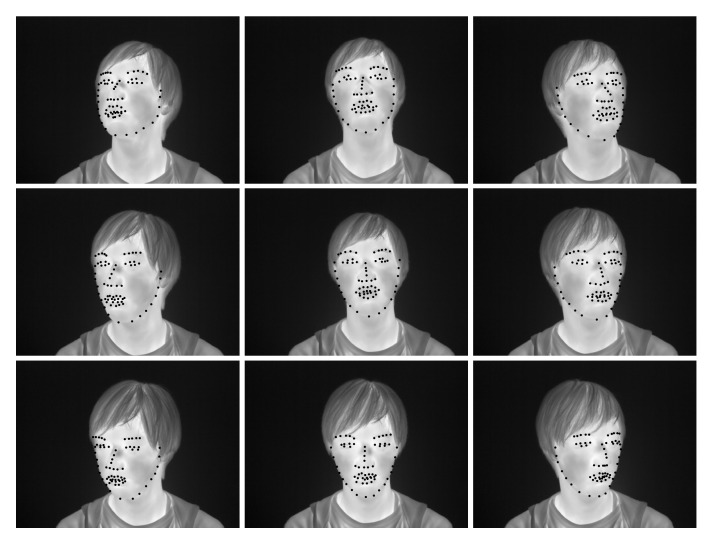
Examples of thermal infrared images oriented in nine different directions.

Experiments were conducted to acquire thermal infrared images of a face for training a facial landmark detector. Seven subjects (five males and two females) aged 22–24 years participated in the experiment. They were fully informed about the experiment and the purpose of the study before their participation. All participants signed a consent form. The experimental system is shown in Figure 1. Thermal infrared images were captured using infrared thermography (FLIR A615-model: A615, 45${}^{\circ }$ field of view, FLIR Systems, Oregon). The infrared camera had a resolution of 640 × 480 pixels and a temperature resolution of less than 0.05 K. Infrared emissivity is the ratio of the thermal radiation from the surface of an object to the radiation from a black body at the same temperature, given by Stefan–Boltzmann’s law. In order to obtain accurate temperature measurements, it is necessary to set the correct infrared emissivity of the surface of an object. In this study, the infrared emissivity of the skin was set to ε = 0.98 [29]. The experimental protocol is shown in Figure 2. Three distances between the subject and infrared thermography were 60 cm, 90 cm, and 120 cm (Figure 3). Each distance consisted of three recording intervals (Small, Large, and Random). As shown in Figure 4, the subjects were asked to turn their heads in nine directions (center, top center, top right, center right, bottom right, bottom center, bottom left, center left, top left) for the Small and Large sections. To evaluate the effect of the angle of face orientation on face alignment, subjects were asked to move their head angles to 20 degrees and 45 degrees in the Small and Large conditions, respectively. To increase the robustness of the face alignment, in the Random section, subjects were asked to move their head in any direction and make any facial expression they wanted. Nothing other than the subject’s body was recorded. The experiment was conducted in the experimental room without convection. Thermal infrared images were taken 15 min after the subjects entered the experimental room for thermal acclimation to the environmental temperature, and the time to take thermal infrared images for each subject was less than 5 min. A total of 609 thermal infrared images were obtained in this experiment. We manually annotated 68 landmarks for the obtained data according to the literature [30] and bounding boxes in the face region.

### 3.2. Analysis Methods

The acquired images were flipped to the left and right for data augmentation. As a result, 1218 images were created. To perform k-fold cross-validation (k = 7) using CSR, we split the data of six subjects into training data and the data of the remaining subjects into test data. All subjects’ data were used as test data. Unless otherwise specified, all experiments were run with the following fixed parameter settings: the number of stages in the cascade T=10, tree depth F=4, number of weak regressors K=500, and a random pair of pixels P=400 used as the difference feature between two points. The average coordinates of the facial landmarks in the training data were used as the initial shape. The Normalized Point to Point Error (NPPE) introduced by Zhu et al. [31] was used as a method to evaluate the estimation accuracy of the face alignment. The $NPPE_{i}$ of each ith image is the following equation:

(4) $$\begin{array}{c} NPPE_{i}=N_{i}\sqrt{\frac{\sum_{n=1}^{N}\left[{\left(x_{n,r}-x_{n,g}\right)}^{2}+{\left(y_{n,r}-y_{n,g}\right)}^{2}\right]}{2N}}, \end{array}$$

(5) $$\begin{array}{cc} & \;N_{i}=\frac{1}{\frac{1}{2}\left(w_{i}+h_{i}\right)} \end{array}$$

where $x_{n,r}$ and $y_{n,r}$ are the coordinates of the estimated facial landmarks, $x_{n,g}$ and $y_{n,g}$ are the coordinates of the correct facial landmarks, *N* is the number of facial landmarks, $w_{i}$ is the width of the face, $h_{i}$ is the height of the face, and $N_{i}$ is the reciprocal of the mean of $w_{i}$ and $h_{i}$. To compare the estimation accuracy of CSR models, we performed Intensity, DSIFT, and HOG-based AAM methods that were effective in aligning faces in thermal infrared images in previous studies [17,22]. Marciniak et al. [32] reported that the accuracy of face recognition in visible images is lower when the number of pixels in the face region is small. To evaluate the effect of the number of pixels in the face region on face alignment, the number of pixels per face width was calculated. To evaluate the computation time, we measured the frames per second (FPS) of the face alignment of the test data for each method. The specifications of the evaluation PC in this experiment were Intel Core i7-8700 CPU and 16GB RAM. Only one CPU core was used. The program was implemented in C++ and Python.

## 4. Results

**Figure 5:**
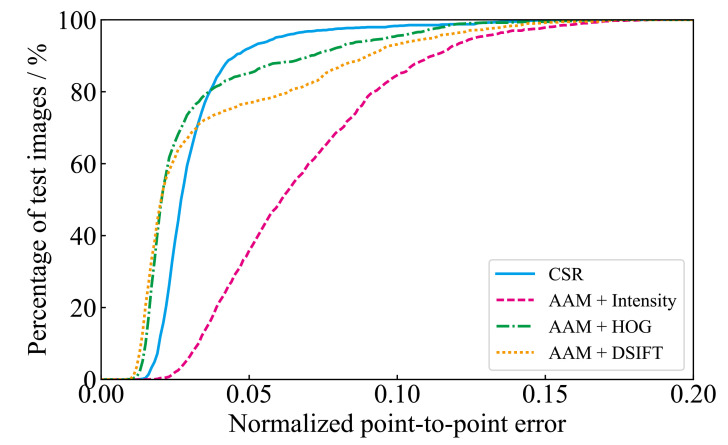
The percentage of test images satisfying a given Normalized Point to Point Error (NPPE) for each method.

**Figure 6:**
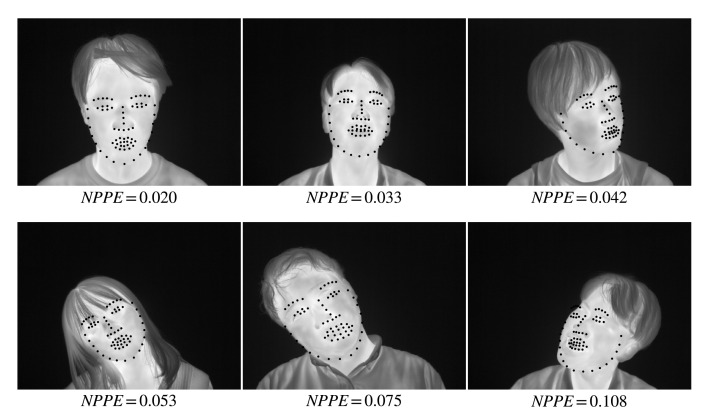
Examples of NPPE for each face alignment.

**Figure 7:**
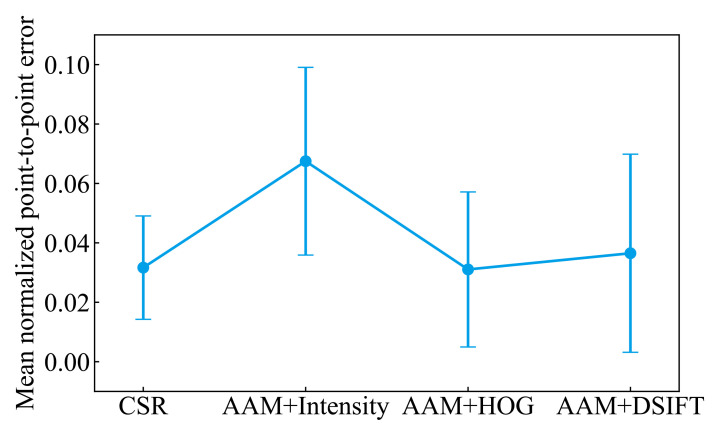
The mean value of NPPE of the test images for each method. Error bars represent standard deviations.

**Figure 8:**
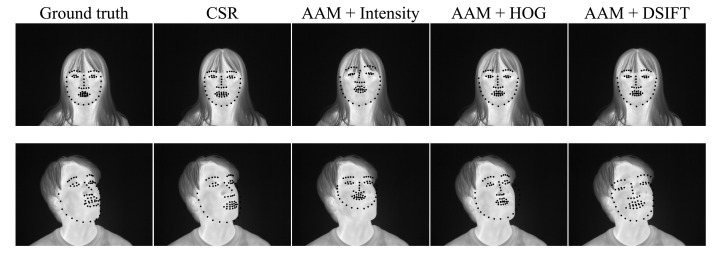
Examples of face alignment using Cascaded Shape Regression (CSR) and Intensity, Dense Scale Invariant Feature Transform (DSIFT), and Histogram of Oriented Gradients (HOG)-based Active Appearance Model (AAM).

Table 1 shows the minimum, maximum, and mean values of the facial skin temperature and the ambient temperature. The ambient temperature was almost the same for all subjects in the experiment. Figure 5 shows the percentage of test images satisfying a given NPPE evaluated with CSR and Intensity, DSIFT, and HOG-based AAM. It is probably due to the problem that AAM is less robust to unknown subjects that are not part of the training set [23]. The CSR method has the highest number of images below 0.05, which is an acceptable accuracy value for NPPE [33]. Figure 6 shows examples of NPPE for face alignment. From Figure 6, it can be confirmed that the accuracy of face alignment becomes worse when the NPPE is greater than 0.5. The CSR method reached a higher total accuracy value. Figure 7 shows the mean value of NPPI of the test images for each method. Figure 8 shows examples of face alignment using each method. The mean NPPEs by CSR and conventional AAM were almost equal. Conversely, the variation of NPPEs was the smallest for CSR. From Figure 8, it can be confirmed that the accuracy of the face alignment of AAM becomes worse when the face is not looking the front. This suggests that face alignment by CSR is more robust than the AAM method and can be applied to face alignment for more varieties of images.

Table 2 shows the FPS of each method: the FPS of the CSR model is over 80, which is more than ten times larger than the AAM methods. The FPS of CSR was the largest, and the FPS of Intensity, DSIFT, and HOG-based AAM were smaller in that order. DSIFT and HOG-based AAMs are considered to have taken more time than Intensity-based AAM because of the calculation of DSIFT and HOG features. It is suggested that face alignment in thermal infrared images using CSR is highly real-time.

**Figure 9:**
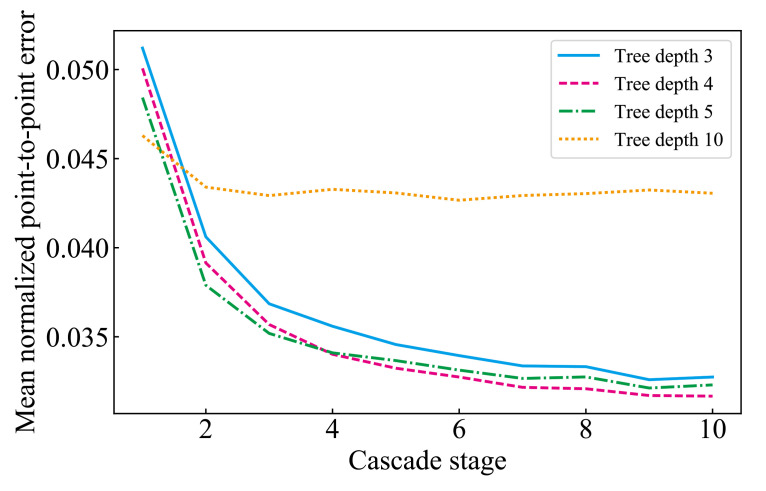
The mean NPPE using CSR for each cascade stage for tree depth = 3, 4, 5, and 10.

**Figure 10:**
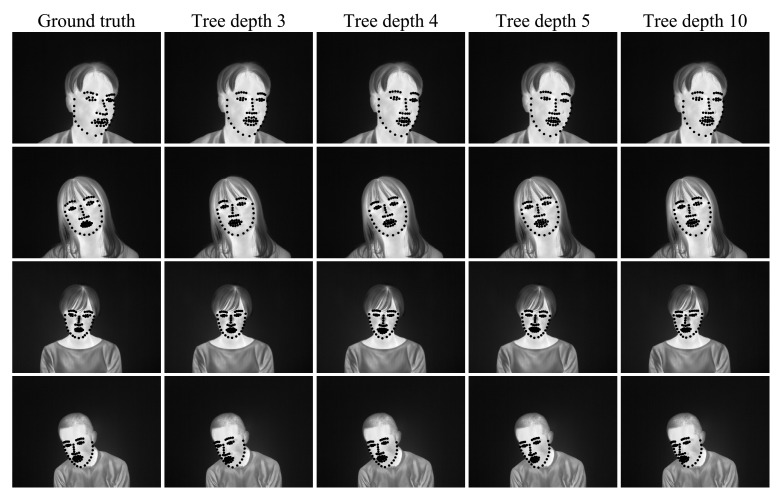
Examples of facial alignment using CSR for each cascade stage for tree depth = 3, 4, 5, and 10.

Figure 9 shows the results for each cascade stage for tree depth = 3, 4, 5, and 10 and Figure 10 shows examples of facial alignment using for tree depth = 3, 4, 5, and 10. When the tree depth was 4, the accuracy of face alignment was the highest. When the tree depth was 5 or 10, the model features were large, and overfitting to the training data occurred, resulting in small accuracy. When the tree depth was 3, the model features were small and under-fitted to the training data, resulting in small accuracy.

**Figure 11:**
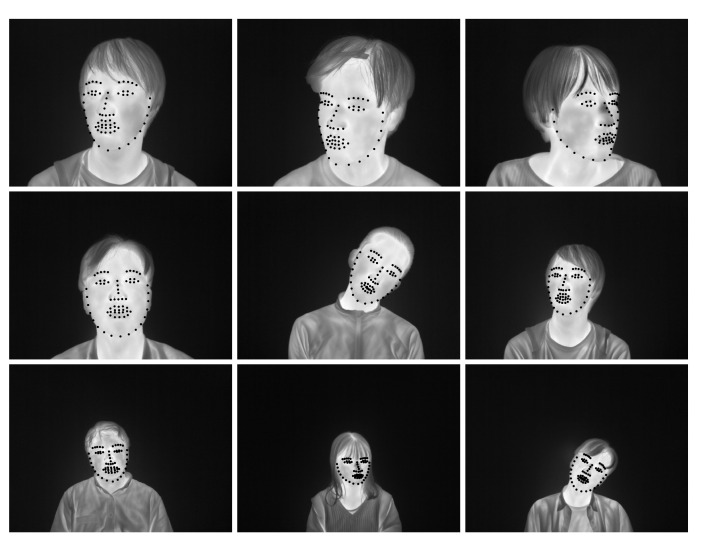
Examples of face alignment for each distance. The distances from the top are 60 cm, 90 cm, and 120 cm.

Table 3 shows the mean number of pixels and mean NPPE of the face’s width for each distance between the infrared thermography and the subject and Figure 11 shows examples of face alignment for each distance. From Table 3 and Figure 11, the accuracy of the fitting did not decrease with distance. In this experimental condition, differences in distance to infrared thermography and the number of pixels of the face in the image did not affect the face alignment estimates’ accuracy. In thermal measurements, one meter is known to be an excellent standard to assure stable consistency [34]. It is suggested that the face alignment can be done with high accuracy when the distance between the thermography and the person is between 60 and 120 cm. This satisfies the length of 1 m, which is the right standard length for thermal measurements.

## 5. Conclusions

As mentioned in the introduction, the objective of this study was to conduct face alignment in thermal infrared images using CSR. CSR is more robust than AAM in face alignment in facial thermal images and can be applied to various types of images. The FPS of face alignment using CSR is more than 80, and it can detect facial landmarks at a high speed. Therefore, facial landmark detection by CSR may be useful for real-world applications. However, the limitation of this study is the small sample size of 609 thermal infrared images, and we have not dealt with thermal infrared images in the wild. In the future, we plan to conduct studies using thermal infrared images of many more varieties and conditions.

## Figures and Tables

**Table 1:** Mean values ± SD of the minimum, maximum, and mean facial skin temperature. The number of thermal infrared images for each subject was 87.

Subject	Facial Skin Temperature/°C			Ambient Temperature/°C
	Minimum	Maximum	Mean	
A	30.64 ± 0.22	34.27 ± 0.15	32.50 ± 0.13	24.42 ± 0.07
B	29.23 ± 0.28	33.76 ± 0.12	31.89 ± 0.22	24.38 ± 0.37
C	29.99 ± 0.20	33.67 ± 0.17	31.85 ± 0.17	24.46 ± 0.14
D	30.81 ± 0.17	34.13 ± 0.17	32.64 ± 0.19	24.02 ± 0.06
E	31.42 ± 0.16	34.18 ± 0.09	32.98 ± 0.09	24.33 ± 0.06
F	30.32 ± 0.33	34.05 ± 0.27	32.27 ± 0.25	24.44 ± 0.29
G	31.20 ± 0.32	34.15 ± 0.17	32.80 ± 0.22	24.11 ± 0.10

**Table 2:** The frames per second (FPS) of each method.

Method	FPS
CSR	83.3
AAM + Intensity	5.56
AAM + HOG	0.35
AAM + DSIFT	0.64

**Table 3:** NPPE using CSR for each distance.

Distance/cm	Pixels Per Face Width	NPPE/%
60	184 ± 20	3.25 ± 1.99
90	126 ± 13	3.02 ± 1.71
120	97 ± 10	3.23 ± 1.47

## Abbreviations

The following abbreviations are used in this manuscript:

AAM	Active Appearance Model
CSR	Cascaded Shape Regression
ROI	Regions of Interest
HOG	Histogram of Oriented Gradients
DSIFT	Dense Scale Invariant Feature Transform
NPPE	Normalized Point to Point Error
FPS	Frames Per Second
